# Effects of endurance exercise and dietary protein intake on osteokine, bone turnover, and inflammatory markers in endurance runners: A narrative review

**DOI:** 10.1016/j.bonr.2025.101850

**Published:** 2025-05-09

**Authors:** Sofia Valente Ferreira, Silar Gardy, Tyler A. Churchward-Venne, Andrea R. Josse, Jenna C. Gibbs

**Affiliations:** a Department of Kinesiology and Physical Education, McGill University, 475 Av des Pins O, Montreal, Quebec H2W 1S4, Canada; b Metabolic Disorders and Complications Program, McGill University Research Institute, McGill University Health Centre, 1001 Decarie Blvd, Montreal, Quebec H4A 3J1, Canada; c Division of Geriatric Medicine, MUHC-Montreal General Hospital,1650 Cedar Avenue, Montreal, Quebec H3G 1A4, Canada; d School of Kinesiology & Health Science, York University, 170 Campus Walk Room 341, Toronto, Ontario M3J 1P3, Canada; e Muscle Health Research Centre, Faculty of Health, York University, Farquharson Life Sciences, 110 Campus Walk, Toronto, Ontario, Canada

**Keywords:** Endurance exercise, Dietary protein, Endurance runners, Osteokines, Bone turnover markers, Inflammatory markers, Running, Bone stress injuries

## Abstract

Bone stress injuries are pervasive among endurance runners due to repetitive sport-specific mechanical loading and a higher prevalence of low energy availability (i.e., inadequate dietary energy intake relative to exercise energy expenditure). Chronic endurance exercise promotes bone formation, thus, runners typically have higher bone mineral density (BMD) than non-weightbearing athletes and sedentary individuals. However, runners may experience increased bone resorption for hours to days following an endurance exercise bout. If recovery is insufficient, uncoupled bone turnover can pose a significant risk to their bone health. While skeletal-immune system crosstalk has been studied, the interaction during and after exercise in athletes is an emerging area of research. Nutritional interventions have been investigated for their effects on bone metabolism surrounding exercise. However, limited research has examined dietary protein intake in endurance athletes, particularly concerning its effects on bone metabolism and osteoimmunology. This narrative review provides an overview of the evidence on the effects of endurance exercise and dietary protein intake on osteokines, bone turnover, and inflammatory markers in endurance athletes. Acute bouts of high-intensity running increase osteokines and bone turnover markers that promote bone resoprtion which parallels increases in pro-inflammatory markers in endurance athletes, suggesting crosstalk between these systems during and after exercise. Chronic endurance exercise promotes increased resting levels of bone formation, while reducing resting pro-inflammatory markers. Adequate dietary protein ingestion habitually and pre-, during, and post-exercise may attenuate bone resportion and pro-inflammatory markers in endurance athletes.

## 1 Introduction

A bone stress injury (BSI) refers to the bone's inability to withstand repetitive mechanical loads, causing microdamage that may lead to stress fractures (Warden et al., 2014). Long-distance runners account for most of the BSIs among various competitive sports and track-and-field athletes (Arendt et al., 2003; Bennell et al., 1996). High or odd-impact sports include high-magnitude loading activities (i.e., jumping, sprinting, accelerating, decelerating, torsional and transverse loads) that transmit high ground reaction forces, creating peak strains on the skeleton that stimulate bone mineral accrual (Tenforde and Fredericson, 2011). For example, gymnastics can produce ground reaction forces >10 times body weight (Hume et al., 2013), while running produces forces 2–4 times body weight (Nilsson and Thorstensson, 1989) which is greater than cycling ground reaction forces (Gatti et al., 2017). Endurance runners apply repetitive mechanical loads on their skeleton during training and competition, which combined with insufficient recovery may disrupt the normal bone remodeling cycle leading to more microdamage formation relative to repair (Warden et al., 2014). A recent meta-analysis and systematic review examining the acute and chronic effects of exercise and nutrition on circulating bone turnover markers in healthy adults found that an acute bout of exercise increases bone resorption measured by increases in carboxy-terminal collagen crosslinks telopeptide of type 1 collagen (CTX), though bone formation measured by procollagen type I N-propeptide (PINP) is often unresponsive or is less pronounced (Dolan et al., 2022; Dolan et al., 2020). Following prolonged, exhaustive exercise, endurance runners also experience elevations in circulating osteokines (signalling molecules, including proteins and peptides, that are secreted by bone cells and other tissues to regulate bone metabolism), specifically sclerostin (Śliwicka et al., 2021), receptor activator of nuclear factor-kappa B ligand (RANKL) (Kerschan-Schindl et al., 2009), and osteoprotegerin (OPG) (Śliwicka et al., 2021; Kerschan-Schindl et al., 2009), favouring bone resorption, which can persist for days into the post-exercise recovery period. Endurance athletes participate in frequent, long-duration training sessions with insufficient recovery, possibly resulting in low-grade systemic inflammation (Cerqueira et al., 2019; da Rocha et al., 2019), which may develop into worse conditions (e.g., overtraining syndrome: a maladapted response to exercise, inability to recover, etc.) and increase the athlete's risk of injury (Smith, 2000). For instance, following a triathlon, endurance athletes sustained low-grade inflammation (i.e., chronic state of low circulating concentrations of inflammatory factors) for 5-days, marked by persisting elevations in circulating pro-inflammatory markers (Neubauer et al., 2008). Combined dietary carbohydrate and protein ingestion post-exercise showed favourable effects on the inflammatory profile in endurance athletes, marked by attenuated pro-inflammatory markers, interleukin-6 (IL-6) and C-reactive protein (Kerasioti et al., 2013). Similarly, Townsend et al. (Townsend et al., 2017) demonstrated beneficial effects of post-exercise dietary carbohydrate and protein co-ingestion on markers of bone turnover (CTX, P1NP) in male endurance runners. However, the impact of dietary protein ingestion alone after exercise on osteokines and inflammatory markers, and its subsequent effect on bone turnover in athletes remains unknown. Previous studies have shown that dietary protein intake promotes increased bone formation markers (i.e., PINP) in young adults (Ballard et al., 2005), and minimizes bone loss in older adults (Hannan et al., 2000). Dietary protein may benefit bone health through various mechanisms such as decreasing bone resorption (Hunt et al., 2009; Theocharidis et al., 2020), stimulating bone collagen synthesis (Babraj et al., 2005), increasing insulin-like growth factor 1 (IGF-1) (Hunt et al., 2009), and increasing lean body mass and muscle strength (Dillon et al., 2009; Børsheim et al., 2008) which can increase muscle contractile forces acting on bone (Nguyen et al., 2020). Thus, dietary protein may provide a prevention strategy for endurance athletes to mitigate the effects of high-training volume and insufficient recovery on bone health.

Nutrition strategies are required for endurance athletes to help mitigate excessive bone resorption induced by their high training demands and optimize their bone recovery and athletic performance. A meta-analysis examining the effects of dietary protein on bone health in healthy adults reported a positive relationship between dietary protein intake and bone mineral density (BMD) and reduced bone resorption markers at rest, with most included studies involving a prescription of dietary protein intake greater than the Recommended Dietary Allowance (RDA) of 0.8 g/kg/day. To our knowledge there are no studies examining the effects of dietary protein intake on sclerostin, RANKL, and OPG in endurance runners. Though, elevated dietary protein intake may be effective at mitigating these bone resorptive responses to exercise based on previously established associations in overweight and obese premenopausal women (Josse et al., 2012). Studies conducted in endurance athletes suggest favourable effects of dietary protein on the acute inflammatory response to exercise, attenuating the post-exercise rise in circulating pro-inflammatory markers (i.e., IL-6 and tumor necrosis factor alpha (TNF-α)) (Kerasioti et al., 2013; Röhling et al., 2021; Rowlands et al., 2008) and increasing anti-inflammatory markers (i.e., IL-10) (Kerasioti et al., 2013; McKinlay et al., 2020). However, the effects of dietary protein on osteokine and inflammatory markers and the downstream effect on bone metabolism in endurance runners remains to be elucidated.

This review provides an overview of the existing research on the effects of endurance exercise, namely running, on osteokine, bone turnover, and inflammatory markers in endurance athletes. Moreover, this review summarizes the current literature on dietary protein intake and supplementation on osteokine, bone turnover, and inflammatory markers in endurance athletes. Studies of acute and chronic endurance exercise with and without dietary protein supplementation and their effects on osteokine, bone turnover, and inflammatory markers will be discussed. However, the focus of this review will be on runners because of the distinct mechanisms of weight-bearing endurance exercise on bone turnover and development of BSIs, though, evidence from other endurance sports (i.e., cycling, swimming) will be discussed because certain topic areas related to our objective have limited research on endurance runners. This review used a manual search strategy of relevant papers/reviews and Boolean operators (AND, OR, NOT) in Google Scholar to broaden the search strategy. The following keywords were used: bone stress injuries (BSIs), runners, athletes, bone, bone health, bone turnover markers, sclerostin, RANKL, RANK, OPG, Wnt/b catenin pathway, RANKL/RANK/OPG pathway, bone formation, bone resorption, inflammation, inflammatory markers, IL-6, TNF-α, IL-10, exercise, post-exercise response, dietary protein, protein supplementation, and other related terms.

## 2 Bone stress injuries in endurance runners

Bone stress injuries are pervasive in endurance athletes due to their high training volume and elevated energetic demands. A BSI occurs along a continuum, beginning with a stress reaction (i.e., weakened bone, bone bruise), then a stress fracture (i.e., small crack), and finally a complete fracture (Warden et al., 2014; Bennell et al., 1999). Arendt et al. (Arendt et al., 2003) conducted a retrospective study reviewing ten years of medical records from Division I college athletes and reported that among 68 males and females with BSIs, specifically stress fractures, long-distance runners accounted for most of these injuries. In a 12-month prospective study in 53 female and 58 male track-and-field athletes, Bennell et al. (Bennell et al., 1996) reported a 21 % incidence rate of stress fractures; 60 % of the athletes who sustained a stress fracture already had a history of at least one fracture, and no differences were observed between males and females. Notably, long-distance runners had the highest percentage of athletes with stress fractures (31.6 %), and middle-distance runners and hurdlers were the subsequent groups most at risk (Bennell et al., 1996). Moreover, among 127 young adult female cross-country runners, about one third had previously experienced at least one stress fracture (Kelsey et al., 2007). The tibia (from the proximal third junction to the distal third junction) was the most common site for stress fractures in athletes, although, other common sites include the navicular, metatarsals, fibula, and femur (Arendt et al., 2003). Though, eumenorrheic athletes who participate in plyometric training have lower rates of BSI prevalence (about 20 %) compared to oligomenorrheic/amenorrhoeic athletes who did (about 25 %) and did not (about 45 %) participate in plyometric training (Hutson et al., 2021). Carbuhn et al. (Carbuhn et al., 2022) reported that out of 79 endurance runners the prevalence of BSIs was 22.8 %, with the injured runners having lower BMD, lean mass (females) or fat mass (males) than noninjured athletes. Therefore, endurance runners are at a higher risk for developing BSIs (e.g., stress fractures) compared to other athletes. However, not all runners will develop BSIs and risk factors such as menstrual disturbances, low BMD, and low lean or fat mass may enhance the risk of developing a BSI.

Early work demonstrated that repetitive mechanical loading resulted in tibia stress fractures in rabbits (Burr et al., 1990), similarly, a loss of stiffness and strength was reported in bovine femurs following repetitive loading (Carter and Hayes, 1977). Mechanical loading (e.g., running) induces bone deformation which is dependent on bone strain (i.e., load magnitude and ability of bone to resist deformation) (Warden et al., 2014). High strains can lead to fractures, although lower strains are capable of initiating microdamage and causing fatigue damage in cortical bone in steers and bovinae (Schaffler et al., 1989; Schaffler et al., 1990). Bone microdamage usually forms at a threshold that depends on bone strain magnitudes, rates, and cycles. Of these factors, load magnitude appears to have the most influence on mechanical fatigue (Loundagin et al., 2018). The absolute threshold is unclear, though once this threshold is surpassed, additional strains can lead to further damage (Warden et al., 2014). Typically, this damage is useful because it acts as a stimulus for targeted remodeling (Burr, 2002). Targeted remodeling maintains the balance between microdamage formation and its repair and increases bone's ability to tolerate loads (Warden et al., 2014). However, endurance runners apply repetitive mechanical loads on their skeleton during sport-specific training/competition, and with insufficient recovery time, this may result in additional microdamage formation. Accelerated bone remodeling, which occurs with loading exercise, may lead to a lag between bone loss and bone formation resulting in an accumulation of microdamage in the weakened bone sites (Warden et al., 2014; Bennell et al., 1999). Microdamage can coalesce into ‘macrocracks’ leading to stress reactions which can result in stress fractures and in some cases, complete fractures (Warden et al., 2014; Frost, 1989).

### 2.1 Bone remodeling

Bone is constructed and rebuilt throughout an individual's lifespan, which is referred to as bone modeling and remodeling, respectively. Bone modeling begins as the skeleton develops as a fetus and continues until the end of skeletal maturity (i.e., approximately at the end of the second decade of life) (Kenkre and Bassett, 2018; Rizzoli et al., 2010). Bone modeling modifies the shape and size of bones through distinct and independent actions of osteoblasts forming bone and osteoclasts resorbing bone (Hughes et al., 2020). In this process, bone resorption and bone formation do not always occur together (i.e., are uncoupled). Instead, they happen at different locations, rates, and times within bone (Kenkre and Bassett, 2018; Rizzoli et al., 2010; Langdahl et al., 2016). Bone remodeling allows the skeleton to repair microdamage (e.g., due to exercise), eliminate old brittle bone, and regulate mineral homeostasis (i.e., namely calcium and phosphorus) (Kenkre and Bassett, 2018). Bone turnover markers are byproducts of proteins or cells released during bone remodeling by osteoblasts and osteoclasts, which reflect systemic levels of bone turnover. Remodeling should be coupled and occurs in both types of bones, although, due to the larger surface area of trabecular bone, it is more metabolically active and undergoes a higher rate of bone turnover than cortical bone. Indeed, the turnover rate of trabecular bone is over 25 % each year, which is much faster than the 2–3 % per year turnover rate of cortical bone (Oftadeh et al., 2015). The remodeling cycle lasts 120 days in cortical bone, and 200 days in trabecular bone (Kenkre and Bassett, 2018).

**Fig. 1:**
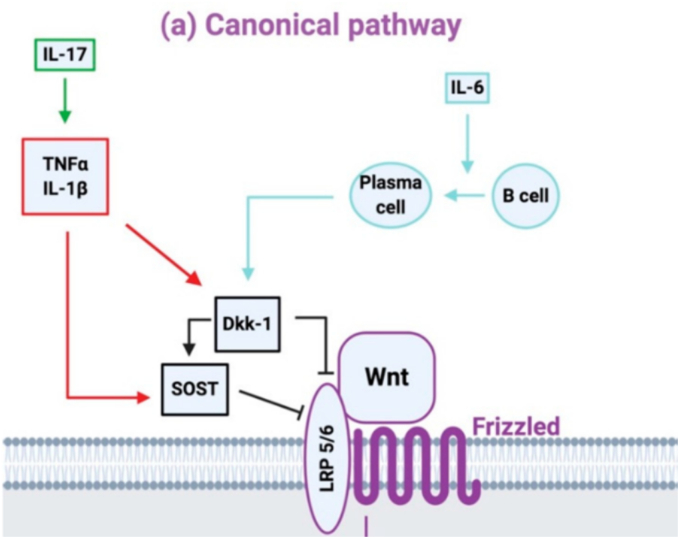
Canonical Wnt signalling pathway with osteokine and pro-inflammatory marker inhibitors. Note. Typically, Wnt proteins bind to their co-receptor complex (Frizzled and low-density lipoprotein receptor-related protein (LRP) 5/6), this triggers a cascade of events resulting in osteoblast differentiation and increased OPG and decreased RANKL. Dickkopf (Dkk-1) and sclerostin (SOST) inhibit this pathway by binding to LRP5/6. Pro-inflammatory markers tumor necrosis factor alpha (TNFα) and interleukin (IL)-1β upregulate Dkk-1 and sclerostin, and IL-6 upregulates Dkk-1. Together, these pro-inflammatory markers inhibit the canonical Wnt signalling pathway via their effects on Dkk-1 and sclerostin, with the downstream effect inhibiting osteoblast differentiation and bone formation. Adapted from Cici D, Corrado A, Rotondo C, Cantatore FP. Wnt signalling and biological therapy in rheumatoid arthritis and spondyloarthritis. International journal of molecular sciences. 2019;20(22):5552 (Cici et al., 2019).

There are numerous molecular pathways involved in the bone remodeling process, although the Wnt/β-catenin and RANKL/RANK/OPG pathways are central for bone formation and resorption, respectively (Kenkre and Bassett, 2018). Interestingly, osteocytes (the most abundant and mature bone cells that regulate bone remodeling) secrete sclerostin (a protein encoded by the *SOST* gene) which inhibits the Wnt/β-catenin signalling pathway, ultimately inhibiting bone formation (Kitaura et al., 2020). Mechanical loading inhibits the production of sclerostin; thus, physical activity and exercise are often negatively correlated with circulating sclerostin concentrations (Oniszczuk et al., 2022). The binding of RANKL to RANK induces bone resorption, although, both osteoblasts and osteocytes release OPG (a decoy receptor for RANKL) to inhibit bone resorption (Kenkre and Bassett, 2018; Kitaura et al., 2020). Various factors, such as parathyroid hormone (PTH), estrogen, and cytokines (e.g., IL-6), can affect either of these two pathways by increasing or decreasing the expression of the *SOST* transcripts and/or sclerostin (see Fig. 1), RANKL, RANK, or OPG proteins. Therefore, normal sclerostin concentrations and an appropriate RANKL:OPG ratio are key components for maintaining balanced bone turnover.

### 2.2 Limitations for bone turnover markers and osteokines

Bone turnover markers are widely used for research and clinical practice to provide insight on the dynamics of bone turnover, which can be a practical tool to further understand metabolic bone diseases and develop treatments, and they are complementary tools to assess fracture risk along with BMD (Schini et al., 2023; Shetty et al., 2016). Bone resorption markers can be measured in the blood and urine, whereas bone formation markers can only be measured in the blood (i.e., serum and plasma) (Schini et al., 2023; Shetty et al., 2016). Bone turnover markers are analyzed via automated or manual immunoassay and multiplex microarrays (e.g., enzyme-linked immunosorbent assays (ELISA)) (Schini et al., 2023; Shetty et al., 2016). These markers provide a timeframe of the bone turnover response in daily living or to a specific stimulus (e.g., nutrition or exercise). However, a few variables should be considered when measuring bone turnover markers, such as, there is inter- and intra-subject variability, they are subject to circadian regulation, most of the markers are not bone-specific (thus making it difficult to render their responses as representative of bone activity alone), their responses may be transient, they are subject to preanalytical and analytical variability (i.e., due to differences in sample collection, storage conditions), and there is a lack of standardization of assays (Dolan et al., 2020; Shetty et al., 2016).

## 3 Acute effects of exercise on bone: an overview

Mechanical loading (i.e., physical activity and exercise) is the primary stimulus for bone formation (Dolan et al., 2020). Following an acute bout of exercise, there is an increase in CTX as a reflection of the normal bone remodeling process (Dolan et al., 2020). Bone formation markers (i.e., PINP) are often unresponsive to an acute bout of exercise (Dolan et al., 2020). Some studies have reported an increase in PINP (Scott et al., 2011; Stunes et al., 2022), although this response is uncommon and less pronounced than that of bone resorption markers. Exercise type, intensity, and duration are key factors for determining the response of bone resorption and formation markers, such that higher exercise intensities typically induce a bone remodeling response, and lower exercise intensities may not (Maimoun et al., 2005). While an acute bout of prolonged cycling has been identified as the aerobic exercise mode with the greatest increases in CTX (Dolan et al., 2022), other types of aerobic exercise, particularly those of low-impact and repetitive nature (i.e., prolonged and/or exhaustive running), warrant further investigation of their effects on bone turnover.

In the following sections, a prolonged run was considered an exhaustive run or a marathon distance or longer, and high-intensity exercise/running was considered as: 1) 15 % above ventilatory threshold; 2) ≥70 % maximal oxygen consumption (VO_2max_); 3) 95 % and/or 110 % anaerobic threshold; or 4) >90 % maximum heart rate.

### 3.1 Acute effects of exercise on osteokines

Athletes have higher circulating sclerostin concentrations than sedentary or recreationally active individuals (Zagrodna et al., 2016), and weight-bearing athletes (i.e., runners) have greater basal sclerostin serum concentrations than non-weight-bearing athletes (Lombardi et al., 2012). This supports the notion that repetitive loading with weight-bearing sports/exercise promotes bone resorption. Athletes' elevated sclerostin concentrations appear to increase with prolonged endurance training and throughout their recovery resulting in prolonged bone resorption. Grasso et al. (Grasso et al., 2015) reported an increase in sclerostin over a 3-week cycling stage race in 9 professional male cyclists, representing sustained elevated bone resorption. Moreover, Śliwicka et al. (Śliwicka et al., 2021) observed elevated sclerostin concentrations 72 h post-marathon in 10 male competitive runners. An increase in sclerostin concentrations during exercise and into recovery among athletes offers a possible explanation for the elevated bone resorption and higher risk for bone loss observed in elite endurance runners. Despite the increase in bone resorption following a high-intensity or prolonged run, OPG concentrations in athletes can remain elevated for 24 h (Scott et al., 2010), 72 h (Śliwicka et al., 2021), and for 3-days after the start of the race (Kerschan-Schindl et al., 2009). Notably, elevated OPG concentrations observed in athletes at the same time may reflect a compensative response to increased bone resorption (i.e., elevated RANKL) (Yano et al., 1999) and/or a mechanism to increase turnover rate to remove damaged bone and replace with new bone.

While there is limited research on RANKL in humans, studies in endurance runners have demonstrated a decrease in circulating RANKL concentrations within 30 min after a marathon (Ziegler et al., 2005) or persistently elevated RANKL concentrations 3 days following the start of an ultramarathon race (Kerschan-Schindl et al., 2009). The discrepancy between these findings may be attributed to the sampling points (i.e., 30 min versus 48 h post-exercise). These findings suggest athletes performing prolonged, exhaustive exercise may have elevated concentrations of bone resorption markers and concomitant increases in OPG for numerous days into recovery. Interestingly, Ziegler and colleagues (Ziegler et al., 2005) did not observe any change in OPG or RANKL concentrations in long-distance runners within 30 min after finishing a middle-distance race (i.e., 15.8 km), though an increase in OPG and decrease in RANKL was observed in those completing a marathon, suggesting that longer distances and high training volumes are required to stimulate this pathway (see Table 1).

**Table 1:** Effects of an acute bout of running on bone turnover, osteokine and inflammatory markers in endurance athletes.

First author, year, ref, country	Participants, sample size	Age, sex, BMI	Methods	Time points of assessments	Outcome/results
Kerschan-Schindl, 2009, (Kerschan-Schindl et al., 2009), Austria	Runners, 16 M^a^ and 2 F^b^, 18 total	43 yrs, M/F, 22.5 kg/m^2^	RM^c^ design. Ultramarathon race (246 km). Wilcoxon's signed rank test.	Blood collected day before race, 15 min after end of race, and 3 days after start of the race.	OPG^d^ and CTX^e^ increased above baseline immediately post-race and remained elevated for 3 days after the start of race. RANKL^f^ increased above baseline 3 days after the start of the race.
Neubauer, 2008, (Neubauer et al., 2008), Austria	Well-trained male triathletes, 42	35 yrs, M, 23 kg/m^2^	RM design. Triathlon. RM-ANOVA^g^.	Blood collected at 2 days pre-race, immediately, 1, 5, 19 days post-race.	IL-6^h^ and C-reactive protein remained elevated above pre-race values for 5 days, and C-reactive protein persisted high for 19 days. IL-10 increased immediately and 1 day post-race and was below pre-race values 5 days post-race.
Scott, 2010, (Scott et al., 2010), United Kingdom	Endurance trained males, 10	29 yrs, M, 24.1 kg/m^2^	Treadmill run to exhaustion at 70 % VO_2max_^i^. Linear mixed model ANOVA.	Blood plasma collected at baseline, during exercise, 2 h post-run, and for 4 days post-exercise.	OPG increased relative to baseline during, 2 h and 1-day post-exercise. No change in PINP^j^, no significant effect of exercise on PINP.
Śliwicka, 2021, (Śliwicka et al., 2021), Poland	Well-trained runners, 10	40.6 yrs, M, N/A	RM design. Marathon race. 1-way RM ANOVA.	Blood collected 24 h before race, 24 h and 72 h after race.	At 24 h and 72 h post-marathon, OPG, TNF-α^k^, and IL-6 were increased above baseline. Sclerostin was increased above baseline 72 h post-marathon.
Zanker, 2000, (Zanker and Swaine, 2000), United Kingdom	Well-trained distance runners, 8	25.1 yrs, M, N/A	Randomized crossover design. 60-min run for 3 days with energy intake balanced or restricted. 2-way RM ANOVA and Student's *t-*test.	Blood collected at baseline and following morning after exercise day (<12 h).	Energy restricted group: PINP decreased in the post-intervention blood draw. Energy balanced group: PINP remained unchanged from pre- to post-intervention.
Ziegler, 2005, (Ziegler et al., 2005), Austria	Long-distance runners, 31	40.5 yrs, M/F, 22.80 kg/m^2^	RM design. Running a marathon or 15.8 km. Wilcoxon Student's paired *t-*test.	Blood collected 30 min before race, and within 30 min after race.	RANKL decreased post-run for all participants. OPG increased post-run only for those completing the marathon.

a Male.

b Female.

c Repeated measures.

d Osteoprotegerin.

e Carboxy-terminal collagen crosslinks telopeptide of type I collagen.

f Receptor activator of nuclear factor-kappa B (RANK) ligand.

g Analysis of variance.

h Interleukin.

i Maximal oxygen consumption.

j Procollagen type I N-propeptide.

k Tumor necrosis factor alpha.

Most of the aforementioned studies did not specify if participants refrained from exercise prior to the study, though these studies included participants completing a marathon or ultramarathon (Śliwicka et al., 2021; Kerschan-Schindl et al., 2009; Ziegler et al., 2005), with the exception of one which did have a rest period for 48 h prior to exercise (Scott et al., 2010). Additionally, not all studies included a fasting protocol, however, as previously mentioned the majority included participating in a marathon or ultramarathon wherein fasting was not possible.

### 3.2 Acute effects of exercise on bone formation and resorption markers

Although discussing the effects of exercise on bone turnover markers is not the primary objective of this paper, this section provides background information on the acute effects of exercise on bone turnover markers. We recommend Dolan et al. (Dolan et al., 2022) for a more comprehensive review on the acute effects of exercise on bone turnover markers.

Endurance-trained male athletes have been reported to have a sustained elevation in circulating CTX concentrations for 4 days following an exhaustive run (Scott et al., 2010). Additionally, endurance-trained males demonstrated elevated CTX concentrations immediately (Maimoun et al., 2005), 1 h (Guillemant et al., 2004), 3 h, and 24 h following high-intensity cycling (Herrmann et al., 2007). No change in CTX concentrations was found following high-intensity cycling in females (Herrmann et al., 2007). Finally, in response to an ultramarathon race, elevated CTX was reported immediately and 3-days post-race in males and females (Kerschan-Schindl et al., 2009). Therefore, an increase in CTX is usually observed in response to an acute bout of exercise, although some studies have reported a decrease or no change, possibly due to the exercise type, limited sampling points, and the transient response of these markers (Dolan et al., 2020).

Bone formation markers, namely PINP, have varying responses to exercise. Numerous studies of high-intensity exercise have reported no change to circulating PINP concentrations in male athletes 24 h after cycling or running (Herrmann et al., 2007; Zanker and Swaine, 2000) and 4-days post-run (Scott et al., 2010) run, and similarly for female athletes at 24 h post-cycling (Herrmann et al., 2007). Furthermore, at moderate intensities (i.e., 75 % anaerobic threshold), PINP concentrations decreased 3 h and 24 h following cycling in male and female athletes (Herrmann et al., 2007). These results regarding the acute effects of endurance exercise on bone formation are inconsistent and the reason for this is unclear (Dolan et al., 2020). Acute endurance exercise appears to have limited effects on PINP, possibly inducing a decrease, although, this response is less common and less pronounced than the bone resorption response (Dolan et al., 2020).

Notably, most of these studies asked participants to refrain from exercise/training for 24–72 h pre-exercise (Maimoun et al., 2005; Scott et al., 2010; Guillemant et al., 2004; Zanker and Swaine, 2000), with only two studies not specifying the pre-exercise protocol (Kerschan-Schindl et al., 2009; Herrmann et al., 2007). Moreover, most participants were in a fasted state for the exercise protocol or blood tests (Maimoun et al., 2005; Scott et al., 2010; Zanker and Swaine, 2000), in two studies the participants were not fasted (i.e., for an ultramarathon race, and testing effect of calcium) (Kerschan-Schindl et al., 2009; Guillemant et al., 2004), and one study did not specify (Herrmann et al., 2007). Thus, it is possible that these differing pre-exercise protocols may have affected the bone turnover marker responses to exercise.

Mechanical loading is thought to induce osteoclast activation and an acute increase in bone resorption, often marked by elevations in CTX, which signals osteoblast activation and bone accrual (Dolan et al., 2020). However, repetitive mechanical stimulation may result in frequent exaggerated bone resorption responses which may outpace formation, resulting in bone fragility in the long-term. Taken together, the evidence supports that endurance athletes may experience an uncoupled bone turnover response to running and cycling (see Table 1), possibly increasing their risk for bone loss and related-injury if this uncoupling is sustained without intervention.

### 3.3 Chronic effects of exercise on bone

Wolff's law states that in healthy individuals, bone will adapt and respond over time to the stress it is placed under, such that, an increase in mechanical loading (i.e., exercise, physical activity) will strengthen both the inner and outer layer of bone (Wolff, 1986). Regular exercise has well-documented long-term osteogenic effects. Most types of exercise will promote bone formation and consequently increase BMD over time (Almstedt et al., 2011; Calbet et al., 2001; Kato et al., 2006; Nickols-Richardson et al., 2007; Vainionpää et al., 2005); hence, runners generally have a greater BMD compared to their non-active counterparts (Bennell et al., 1997; Brahm et al., 1997), and to non-weightbearing endurance athletes (i.e., cyclists and swimmers). Niinimäki et al. (Niinimäki et al., 2019) reported that endurance runners, soccer and squash players, and high and triple jumpers had greater mid-shaft femoral bone geometry properties (i.e., cortical and total area, bending and torsional rigidity) than nonathletes and swimmers, suggesting that bone strains induced by ground reaction forces elicit greater osteogenic effects than muscle tensile forces (Niinimäki et al., 2019; Duncan et al., 2002).

Due to their high training volumes, athletes often have altered osteokine, bone turnover and inflammatory markers at rest and following exercise after years of training. Elite female rowers training over an Olympic year demonstrated sclerostin concentrations that fluctuated parallel to training load (product of training volume and intensity), thus, reaching its peak during the week with the highest training load (22 h/week) (Kurgan et al., 2018). A similar sclerostin response to exercise has previously been reported in male cyclists who had an increase in sclerostin concentrations over a 3-week long cycling race (Grasso et al., 2015). Moreover, sclerostin increased parallel to pro-inflammatory cytokines, such as IL-6, and more so with TNF-α, suggesting that athletes' elevated training volumes may inhibit bone formation by upregulating sclerostin and pro-inflammatory markers (Kurgan et al., 2018). Joro and colleagues (Joro et al., 2017) noted that national-level athletes with overtraining syndrome had greater pro-inflammatory responses (i.e., IL-1β) following a cycling exercise to volitional exhaustion at 6 and 12 months, whereas healthy athletes had elevated anti-inflammatory responses (i.e., IL-10) at these time points. These results suggest that excessive training loads in athletes may have detrimental effects on bone health indirectly through elevated inflammation. Interestingly, in elite female rowers, RANKL was found to decrease over a year and OPG stabilized, thus increasing the OPG:RANKL ratio and suggesting a protective mechanism against the effect of high-training volume on bone metabolism (Kurgan et al., 2018).

## 4 Immune and skeletal system crosstalk

Crosstalk between the immune and skeletal system has been studied since the 1970s (Horton et al., 1972), and has given rise to a new field termed osteoimmunology (Takayanagi, 2007). The interaction between these two systems is complex, although, the most typical interaction is observed in pathological states of the immune system leading to bone destruction (Takayanagi, 2007); and inflammation can directly and indirectly act on bone (Hardy and Cooper, 2009).

Inflammation is the body's generalized response to tissue damage, regardless of the cause, and exercise functions as a stressor that induces local and possibly chronic inflammation (Smith, 2000). Following exercise, there is an acute increase in inflammatory markers (i.e., TNF-α, IL-6, IL-1β, IL-10), which may persist for hours and even days into recovery, reflecting the body's response to tissue damage and repair (Ostrowski et al., 1999; Starkie et al., 2001). In general, these inflammatory cytokines acutely increase to a greater extent with high- rather than moderate-intensity exercise (Cerqueira et al., 2019). Notably, IL-6 consistently increases more than other cytokines, and TNF-α and IL-1β are only stimulated by high-intensity exercise (Cerqueira et al., 2019; Ostrowski et al., 1999). Furthermore, long-term exercise has a beneficial effect on resting inflammatory cytokines, such that physically active individuals have lower pro-inflammatory markers than sedentary individuals (Colbert et al., 2004; Panagiotakos et al., 2005; Mikkelsen et al., 2013; Aguiar et al., 2020; Rittweger et al., 2024).

Exercise-induced acute inflammation may eventually result in chronic and possibly systemic inflammation as a result of persistent high volume/intensity training with limited rest (Smith, 2000). When athletes participate in frequent, high-intensity and/or prolonged exercise combined with insufficient rest resulting in an elevated and sustained inflammatory response leading to systemic low-grade inflammation (Cerqueira et al., 2019; da Rocha et al., 2019), which may develop into overtraining syndrome. Approximately 60 % of long-distance runners are classified as overtrained (i.e., excessive training with performance deterioration that induces a local acute inflammatory response), which may result in chronic and systemic inflammation (Smith, 2000). Neubauer and colleagues (Neubauer et al., 2008) reported that endurance athletes sustained low-grade inflammation for 5-days following an intensive endurance race (i.e., triathlon) based on elevated circulating concentrations of IL-6 and high-sensitive C-reactive protein (see Table 1). Therefore, frequent, high-intensity training, coupled with incomplete recovery between training sessions, may have deleterious effects on athletes' health and performance as a result of these inflammatory responses.

### 4.1 Inflammatory markers TNF-α, IL-6, IL-10 and bone cells

The numerous mechanisms by which inflammatory markers (i.e., TNF-α, IL-6, IL-10) interact with bone cells extend beyond the scope of this review; thus, only proposed mechanisms in endurance runners will be discussed.

In vivo, animal, and clinical studies show that TNF-α may act on osteocytes to increase sclerostin and RANKL expression (Gharbia et al., 2020; Baek et al., 2014; Kim et al., 2012; Ohori et al., 2019), ultimately stimulating osteoclast formation and bone resorption (Ohori et al., 2019). We propose a similar interaction with endurance exercise, because following a high-intensity cycling and running bout in recreationally active young adults, TNF-α strongly predicted sclerostin's response (Kouvelioti et al., 2019). Moreover, TNF-α has been associated with sclerostin fluctuations over an Olympic year in elite female rowers (Kurgan et al., 2018). Therefore, TNF-α may play a crucial role in the elevated bone loss observed in certain athletes, specifically via its role on osteocyte-mediated bone resorption and elevated sclerostin expression.

Interleukin-6 is a key regulator of the acute phase response of inflammation (i.e., systemic response to disturbances in homeostasis caused by infection, tissue damage, trauma, or surgery) (Castell et al., 1989), in which it has a dual role as a pro- and anti-inflammatory marker (Gabay, 2006). Typically, IL-6 is produced by osteoblasts to induce osteoclast activity and promote bone resorption (Ishimi et al., 1990), though in vitro studies have shown that IL-6 directly inhibited the differentiation of osteoclast precursors via the RANKL signalling pathways (Yoshitake et al., 2008). A recent in vitro study reported that these discrepancies in the regulation of osteoclasts by IL-6 may be explained by varying concentrations of RANKL in murine cells (Feng et al., 2017). Therein, when RANKL concentrations are high, IL-6 with soluble IL-6 receptor inhibits osteoclast formation and bone resorption, and when RANKL concentrations are low, the opposite occurs (Feng et al., 2017). The blockade and infusion of IL-6 in humans showed no effect on CTX and PINP, however, the assessments of these bone turnover markers were explorative (Lehrskov et al., 2020). The evidence for the effects of IL-6 on osteoclastogenesis and bone resorption remains controversial and human data is scarce, although, findings favour enhanced bone resorption, especially under pathological conditions, such as in individuals with rheumatoid arthritis (Kotake et al., 1996). Therefore, IL-6 may partly regulate the elevated bone resorption reported in athletes by directly and indirectly stimulating osteoclasts, possibly when RANKL concentrations are low. Indeed, regular exercise has an overall positive anti-inflammatory effect (Aguiar et al., 2020; Mattusch et al., 2000; Petersen and Pedersen, 2005; Thompson et al., 2010), however, prolonged moderate-to-high-intensity exercises (i.e., >60 % VO_2max_, >60 % or 76 % heart rate maximum, Borg rating of perceived exertion scale >13) with diminished recovery periods may result in dysregulated immune system responses (i.e., elevated pro-inflammatory markers) (Cerqueira et al., 2019). Thus, endurance athletes may more susceptible to this immune response dysregulation due to their high training demands. Insufficient recovery periods following repeated or prolonged bouts of exercise may induce sustained inflammation and attenuate the anti-inflammatory effect of exercise (Neubauer et al., 2008; Sahl et al., 2017; Suzuki et al., 1999), possibly resulting in overtraining syndrome and increased risk of injury and illness (Smith, 2000).

Anti-inflammatory IL-10 has a potent inhibitory effect on osteoclastogenesis by directly inhibiting the differentiation of osteoclast precursors into osteoclasts in bone marrow cells of mice and rats (Lovibond et al., 2003; Xu et al., 1995). Interleukin-10 promotes bone formation by enhancing human mesenchymal stem cells osteogenic activity (Vallés et al., 2020), and increasing mineralization in vivo and *vitro* mouse models (Xiong et al., 2020). Notably, anti-inflammatory IL-10 also inhibited the production of various inflammatory cytokines in human monocytes, such as IL-6, IL-1β, and the greatest inhibitory effect was on TNF-α (de Waal Malefyt et al., 1991). Therefore, IL-10 demonstrates favourable effects on bone health by direct and indirect actions on osteoclasts, osteoblasts, and other inflammatory markers.

### 4.2 Inflammatory marker and osteokine interactions with exercise

**Fig. 2:**
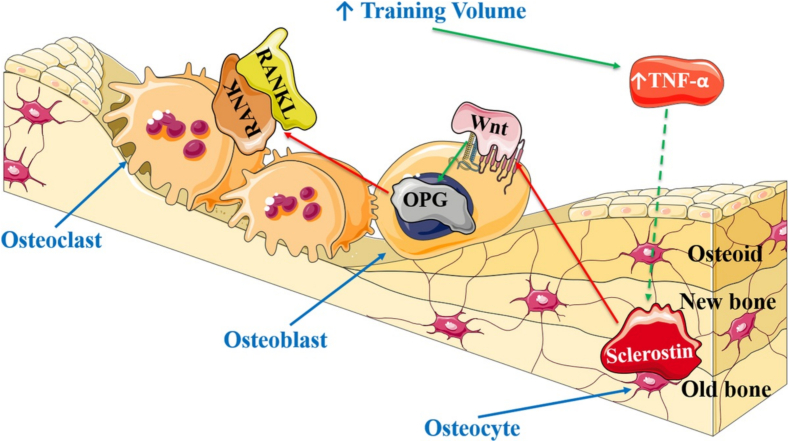
The proposed effect of endurance training volume on Wnt/β-catenin and RANK/RANKL/OPG signalling. Note. A similar mechanism of action is proposed for IL-6 in response to increased training volume. Effects of increased training volume of TNF-α and IL-6 could also directly affect OPG and RANKL. Green solid lines demonstrate well-documented relationships for the activation or increased expression of a protein; green dotted line indicates a potential explanation for elevated sclerostin; red solid lines represent well-defined inhibitory relationships. OPG = osteoprotegerin; RANKL = receptor activator of nuclear factor-kappa B (RANK) ligand; TNF-α = tumor necrosis factor alpha. From Kurgan N, Logan-Sprenger H, Falk B, Klentrou P. Bone and Inflammatory Responses to Training in Female Rowers over an Olympic Year. Medicine & Science in Sports & Exercise. 2018;50(9):1810–7 (Kurgan et al., 2018).

**Fig. 3:**
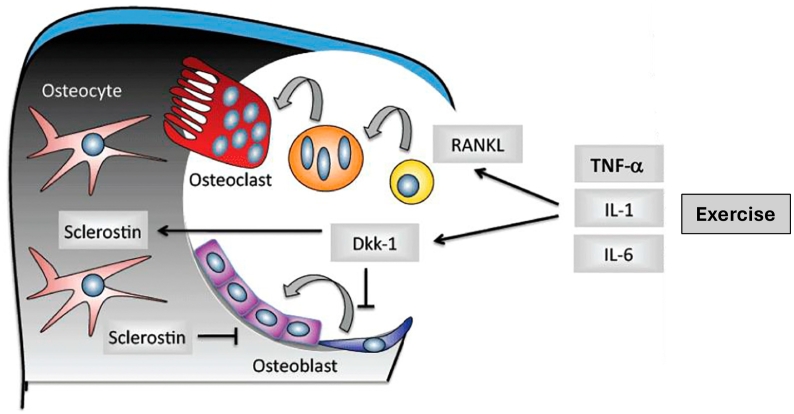
Acute effects of exercise-induced inflammation on Wnt/β-catenin and RANK/RANKL/OPG signalling pathways. Note. High-intensity and/or prolonged exercise increases pro-inflammatory markers tumor necrosis factor alpha (TNF-α) and interleukin-6 (IL-6) and IL-1. These pro-inflammatory markers increase expression of receptor activator of nuclear factor-kappa B ligand (RANKL) which promotes osteoclast differentiation and bone resorption, while also upregulating Dickkopf (Dkk-1) and sclerostin which inhibit bone formation. Ultimately, these mechanisms may promote exercise-induced bone loss, especially in athletes. Adapted from Lange U, Dischereit G, Neumann E, Frommer K, Tarner I, Müller-Ladner U. Osteoimmunological aspects on inflammation and bone metabolism. J Rheum Dis Treat. 2015;1(008) (Lange et al., 2015).

Despite a mechanistic understanding of the interactions between inflammatory markers and osteokines in animal models, less is known about these relationships in healthy adults, particularly in response to exercise. Zhao et al. (Zhao et al., 2024) provide an extensive review on the role of the immune system on bone remodeling and homeostasis and the role of exercise in osteoimmunology that goes beyond the scope of this review. Kurgan and colleagues (Kurgan et al., 2018) concluded that in elite endurance athletes, sclerostin fluctuates parallel to IL-6 and even more so with TNF-α throughout a competitive season, such that training periods of high volume induce inflammation and inhibit osteoblasts (i.e., elevated sclerostin, TNF-α, and IL-6) (see Fig. 2). These findings suggest that in athletes, sclerostin correlated with inflammatory markers, particularly TNF-α and IL-6, during exercise (Kurgan et al., 2018). Thus, markers of systemic inflammation may partly regulate the exercise-induced bone remodeling response via the regulation of sclerostin. In another study by Śliwicka et al. (Śliwicka et al., 2021), endurance athletes were reported to have elevated circulating TNF-α and IL-6 concentrations for 24 and 72 h following a marathon, demonstrating that inflammation persists following prolonged, exhaustive exercise in athletes (see Table 1). The increase in TNF-α paralleled that of OPG at 24 and 72 h post-marathon, suggesting that OPG may increase as a protective mechanism to suppress RANKL activity and inhibit exercise-induced inflammation (Śliwicka et al., 2021). A positive correlation between IL-10 and OPG has also previously been reported, indicating that IL-10, an anti-inflammatory marker, reduces bone resorption possibly by influencing OPG (Mezil et al., 2015). Therefore, elevated bone resorption in endurance runners may be partly attributed to inflammatory markers and their regulatory roles on sclerostin, RANKL and OPG (see Fig. 3).

## 5 Nutrition on bone health

Bone is a nutritionally regulated organ. Key nutrients involved in maintaining optimal bone health include protein, calcium, and vitamin D (Mitchell et al., 2015; Palacios, 2006). Protein makes up about 50 % of bone's volume and one third of its mass (Sale and Elliott-Sale, 2019; Tsagari, 2020). Greater dietary protein intake has positive effects on the skeleton by increasing intestinal calcium absorption, suppressing PTH, increasing IGF-1, and enhancing muscle mass and strength (Kerstetter et al., 2011). Dietary protein intake provides the amino acids necessary to (re)build the bone matrix via protein turnover and stimulates IGF-1 production (Mitchell et al., 2015; Palacios, 2006). The organic extracellular matrix of bone is mostly (90 %) composed of type 1 collagen, which is abundant in glycine, proline, and hydroxyproline (Naomi et al., 2021). Shaw et al. (Shaw et al., 2017) supplemented recreationally active young men with 0, 5, or 15 g of vitamin C-enriched gelatin (a food derivative of collagen) throughout an intermittent exercise program, and found increased collagen content and PINP, suggesting that gelatin in humans enhances collagen synthesis following exercise (Shaw et al., 2017). However, numerous studies since Shaw et al. have demonstrated opposing results, wherein collagen supplementation had no effect on bone turnover marker responses to exercise in young recreationally active adults (Clifford et al., 2019; Hilkens et al., 2023; Aussieker et al., 2023). Aussieker et al. (Aussieker et al., 2023) reported that in recreational athletes, collagen and whey protein ingestion following a resistance training exercise inhibited the increase in CTX, thus whole body collagen breakdown, compared to a noncaloric placebo. Furthermore, intravenous feeding of amino acids, triglycerides, and glucose, stimulated a marked increased (∼66 %) in bone collagen synthesis in young healthy men (Babraj et al., 2005). Long-term high-protein diets or supplementation favour increased circulating IGF-1 in postmenopausal women (Hunt et al., 2009; Zhu et al., 2011), older men (Khalil et al., 2002), and children (Hoppe et al., 2004). A meta-analysis examining the influence of nutrition interventions on bone turnover markers to acute bout of exercise, concluded that dietary protein or carbohydrate supplementation may attenuate the CTX response to exercise (Dolan et al., 2024), though this analysis was not confined to athletes. Considering protein's critical role in bone health, there is limited experimental research, especially among athletes, on the effects of dietary protein alone on bone metabolism. This may be due to the previously proposed ‘acid-ash hypothesis’ by Wachman and Bernstein (Wachman and Bernstein, 1968), stating that elevated dietary protein intakes, especially animal protein (i.e., such as with a Western diet), has a negative effect on bone health. However, Kerstetter and colleagues (Kerstetter et al., 2005) later discovered that a high protein diet increased intestinal calcium absorption which paralleled the increase in urinary calcium excretion.

Energy availability is a central component for regulating bone health in athletes and low EA can be achieved through reduced dietary energy intake, increased exercise energy expenditure, or a combination of both. Notably, diet is the primary factor responsible for disruptions in bone metabolism stimulated by low EA. In active young eumenorrheic females, diet-induced low EA reduced bone formation significantly (Papageorgiou et al., 2018), whereas exercise-induced low EA did not affect bone metabolism (Papageorgiou et al., 2018), suggesting that the osteogenic effects of mechanical loading may have counteracted the bone loss induced by low EA. Therefore, adequate dietary energy intake is pertinent for endurance athletes to maintain healthy bones and peak performance. Bone turnover markers have a circadian rhythm, specifically, bone resorption markers are highest at night and lowest in early afternoon (Wichers et al., 1999). The circadian rhythm of bone resorption markers appears to be primarily modulated by feeding patterns (Schlemmer and Hassager, 1999). Feeding induces a greater variation in the bone resorption circadian rhythm marked as a greater decrease in bone resorption markers, and fasting promotes bone resorption by reducing the fluctuation in bone resorption markers which highlights the importance for measuring individuals in a fasted stated to reduce the individual variability in these markers (Schlemmer and Hassager, 1999). Previous studies have demonstrated that a breakfast meal (self-selected, or 60 % carbohydrates, 32 % fat, 8 % protein, 116 mg calcium, or 75 g glucose, 35 g fat, 40 g protein) suppresses bone resorption markers and to a lesser extent bone formation markers at rest (Clowes et al., 2002; Henriksen et al., 2003; Scott et al., 2012), and during exercise (Scott et al., 2012).

### 5.1 Dietary protein interventions in athletes

Dietary protein has well-documented effects on post-exercise muscle recovery, especially by increasing muscle protein synthesis (Reidy and Rasmussen, 2016; Breen et al., 2011; Churchward-Venne et al., 2020) and muscle mass and strength (Cermak et al., 2012). Dietary protein is not typically considered an efficient fuel source for exercise. However, studies in endurance athletes show that when co-ingested with carbohydrates, protein can provide benefits during prolonged endurance exercise by improving net protein balance and reducing markers of muscle damage (Koopman et al., 2004; Luden et al., 2007). Furthermore, two studies have reported an attenuation of post-exercise bone resorption in athletes consuming protein (Theocharidis et al., 2020) or a combined protein and carbohydrate beverage (Townsend et al., 2017). However, the mechanisms of the potential effects of dietary protein on bone during or after endurance exercise remain unknown. At rest, dietary protein may exert its effects on bone by suppressing pro-inflammatory markers (Hruby and Jacques, 2019) and PTH (Kerstetter et al., 1997), and increasing calcium absorption (Hunt et al., 2009; Kerstetter et al., 2005), IGF-1 (Hunt et al., 2009), collagen synthesis (Shaw et al., 2017), muscle protein synthesis (Moore et al., 2014), and lean body mass with resistance exercise training (Nunes et al., 2022). Therefore, dietary protein may offer a potential nutritional strategy to improve bone health and mitigate BSI risk in athletes, though further research is required to address this knowledge gap.

There is limited evidence on the effects of dietary protein intake on sclerostin, RANKL, and OPG in endurance runners. Though, in pre-menopausal females, a high protein diet (i.e., 30 % of daily energy from protein) reduced resting RANKL and increased resting OPG (Josse et al., 2012). On the other hand, in adult males, a high protein diet of 1.7 g/kg/d of body weight demonstrated no significant effect on sclerostin following an acute bout of exercise, though, this study was not powered to detect this change (Murphy et al., 2021). Therefore, further research is required to understand the effects of dietary protein on osteokines in athletes.

A systematic review concluded that the research on the effects of protein ingestion on post-exercise inflammation in young healthy adults is inconsistent (i.e., some studies show no effects on inflammatory or oxidative stress markers and others showing effects of anti-inflammatory or antioxidant markers). Although, just over half of the studies included in this review were conducted in athletes or trained individuals (i.e., 19 out of 34) (Alhebshi et al., 2022). A study with male cyclists showed that the ingestion of carbohydrates and protein (0.9 g carbohydrate/kg body weight/hour and 0.26 g protein/kg body weight/hour) together following a high-intensity cycling trial (i.e., to exhaustion) attenuated the post-exercise inflammatory response by decreasing IL-6 (Kerasioti et al., 2013). A study in male and female competitive adolescent swimmers reported that the consumption of whey protein (0.3 g/kg body mass) following high-intensity swimming (i.e., maximal effort) increased IL-10 during recovery more so than the water/placebo group (McKinlay et al., 2020). Moreover, 3-months of protein supplementation (skim milk and soy protein, 53.3 % protein content) attenuated the rise in IL-6 following a marathon race in endurance trained adults compared to the control group who did not receive supplementation (Röhling et al., 2021). Six-weeks of whey protein supplementation (40 g) among young female endurance runners did not significantly decrease pro-inflammatory markers (i.e., C-reactive protein and IL-1) following a 1-hour run (Tara et al., 2013). A high-carbohydrate-protein (with fat) (1.4 g/kg/h carbohydrate, 0.7 g/kg/h protein, and 0.26 g/kg/h fat) recovery feeding showed a trend (*p* = 0.055) to reduce TNF-α and IL-6 during recovery from a high-intensity cycling trial (i.e., 80–90 % peak power output) (Rowlands et al., 2008). The small sample sizes of these two studies (*n* = 18 and 12, respectively) may have limited the statistical power to detect significant effects (Rowlands et al., 2008; Tara et al., 2013). Although the general consensus from systematic reviews and meta-analyses was that protein supplementation had no beneficial effects on inflammatory markers (Alhebshi et al., 2022; Akbari et al., 2023), studies conducted in athletes or highly active individuals suggest a favourable effect on the inflammatory response to exercise (i.e., attenuated pro-inflammatory markers and/or increased anti-inflammatory markers) (Kerasioti et al., 2013; Röhling et al., 2021; Rowlands et al., 2008; McKinlay et al., 2020; Tara et al., 2013).

Dietary protein may also affect bone turnover during and after exercise among athletes, which may contribute to their long-term bone health and reduce the risk of BSIs. Notably, among adolescent competitive swimmers, the consumption of 0.3 g/kg of whey protein following a maximal effort (i.e., determined by their split pace for a 200 m maximal speed swim) swimming trial led to a delayed decrease in CTX compared to the groups that ingested carbohydrates or water with no observed change in PINP in any groups (Theocharidis et al., 2020). Furthermore, in male endurance runners, the immediate and delayed ingestion of a combined protein and carbohydrate beverage (1.5 g/kg body mass dextrose and 0.5 g/kg body mass whey protein) suppressed CTX more than the placebo beverage (water, taste-matched) throughout the recovery period following an exhaustive run at 75 % VO_2max_ (Townsend et al., 2017). The placebo beverage was not isocaloric, thus, it is unknown if this response was due to the act of feeding or the effects of carbohydrates and protein, though this does show proof of principle that this supplement may mitigate bone resorption responses. Therefore, post-exercise protein supplementation among endurance athletes, may attenuate bone resorption throughout recovery. Evidence for pre-exercise protein supplementation and/or a high-protein meal and its effects on bone turnover markers is mixed. Among pre-pubertal gymnasts, a pre-training high-carbohydrate meal (88 % carbohydrates, 9 % protein, 3 % fat) attenuated the rise in CTX more than a high-protein meal (55 % carbohydrates, 31 % protein, 13 % fat) (Amato et al., 2020). Furthermore, a calcium-rich dairy meal (1352 mg) ingested prior to a prolonged cycling session attenuated the rise of CTX compared to the control group (ingested an isocaloric meal), and with no effect on PINP, in 32 female competitive cyclists (Haakonssen et al., 2015). Though this study included a calcium-rich dairy meal, dairy products such as milk and yogurt were included which are high in casein and whey protein (Haakonssen et al., 2015). Therefore, it is plausible that pre-exercise protein intake may attenuate bone resorption in endurance athletes, although further research is required.

## 6 Conclusion

Endurance athletes, particularly runners, are at a higher risk of BSIs due to repetitive sport-specific mechanical loading and a higher prevalence of low energy availability. The current literature indicates that acute high-intensity endurance exercise leads to a sustained increase in sclerostin and possibly RANKL that persists for several days into recovery, a similar response is observed in bone resorption marker CTX while bone formation markers like PINP often remain unchanged. In endurance athletes, fluctuations in inflammatory markers parallel those of sclerostin and OPG during and after exercise, suggesting that exercise-induced inflammation may influence athletes' bone metabolism through various osteokines. Chronic endurance training in endurance athletes may result in elevated resting sclerostin and bone formation markers, minimal/no change in bone resorption markers, and reduced pro-inflammatory markes. However, these results are less well-supported than the acute responses and longitudinal studies are required. Dietary protein intake/supplementation may offer a promising nutritional strategy to mitigate the unfavorable bone turnover, osteokine, and inflammatory responses to endurance exercise in athletes. However, we first need to conduct experimental studies to test the effects of dietary protein on bone turnover, osteokine, and inflammatory responses to exercise in endurance athletes relative to controls and other nutritional supplements. This review highlights the necessity for research on the interactions between bone metabolism and inflammation in endurance athletes, and specifically with the effects of dietary protein as a nutrition intervention. Gaining a better understanding of these interactions are necessary to identify optimal exercise and/or nutrition interventions to improve bone health while maintaining performance among endurance athletes.

## Abbreviations

BSI: bone stress injury
CTX: carboxy-terminal collagen crosslinks telopeptide of type 1 collagen
PINP: procollagen type I N-propeptide
RANK: receptor activator of nuclear factor-kappa B
RANKL: receptor activator of nuclear factor-kappa B
OPG: osteoprotegerin
BMD: bone mineral density
IL: interleukin
TNF-α: tumor necrosis factor alpha
EA: energy availability
PTH: parathyroid hormone
VO_2max_: maximal oxygen consumption

## Ethics approval and consent

Not applicable.

## CRediT authorship contribution statement

**Sofia Valente Ferreira:** Writing – review & editing, Writing – original draft, Visualization, Validation, Resources, Methodology, Data curation, Conceptualization. **Silar Gardy:** Writing – review & editing. **Tyler A. Churchward-Venne:** Writing – review & editing. **Andrea R. Josse:** Writing – review & editing. **Jenna C. Gibbs:** Writing – review & editing, Conceptualization.

## Funding

This research did not receive any specific grant from funding agencies in the public, commercial, or not-for-profit sectors. JCG is the recipient of a Fonds de recherche du Québec (FRQS) Chercheurs-boursiers Research Scholar Award (Junior 1).

## Declaration of competing interest

The authors declare no competing interest.

## Data Availability

No data was used for the research described in the article.
